# Image-Processing-Based Low-Cost Fault Detection Solution for End-of-Line ECUs in Automotive Manufacturing

**DOI:** 10.3390/s20123520

**Published:** 2020-06-22

**Authors:** Adrian Korodi, Denis Anitei, Alexandru Boitor, Ioan Silea

**Affiliations:** Faculty of Automation and Computers, Department of Automation and Applied Informatics, University Politehnica Timisoara, 300223 Timisoara, Romania; anitei_denis@yahoo.com (D.A.); b.xanderr@gmail.com (A.B.); ioan.silea@upt.ro (I.S.)

**Keywords:** automated optical inspection, automotive manufacturing, fault detection, image processing

## Abstract

The manufacturing industry is continuously researching and developing strategies and solutions to increase product quality and to decrease production time and costs. The approach is always targeting more automated, traceable, and supervised production, minimizing the impact of the human factor. In the automotive industry, the Electronic Control Unit (ECU) manufacturing ends with complex testing, the End-of-Line (EoL) products being afterwards sent to client companies. This paper proposes an image-processing-based low-cost fault detection (IP-LC-FD) solution for the EoL ECUs, aiming for high-quality and fast detection. The IP-LC-FD solution approaches the problem of determining, on the manufacturing line, the correct mounting of the pins in the locations of each connector of the ECU module, respectively, other defects as missing or extra pins, damaged clips, or surface cracks. The IP-LC-FD system is a hardware–software structure, based on Raspberry Pi microcomputers, Pi cameras, respectively, Python and OpenCV environments. This paper presents the two main stages of the research, the experimental model, and the prototype. The rapid integration into the production line represented an important goal, meaning the accomplishment of the specific hard acceptance requirements regarding both performance and functionality. The solution was implemented and tested as an experimental model and prototype in a real industrial environment, proving excellent results.

## 1. Introduction

With the application and promotion of Industry 4.0 in industrial engineering, each big manufacturing plant received obvious improvements in human cost, quality-enhancing, rapid customization, and manufacturing, and learned that the application of advanced intelligent processing technology to manufacturing is the new development trend. As stated in [1], improving the yield rate and reducing manufacturing costs involves the use of inspection devices, which is widespread in all steps of the production lines, such as design, layout, manufacturing, assembly, and testing. The Automated Optical Inspection (AOI) has attracted increasing interest in product quality control in both academic and industrial communities, particularly on mass production processes, because product quality can often be characterized by their corresponding surface visual attributes. Considering that most product qualities can be characterized by corresponding surface visual attributes, the visual appearance, including the attributes of color, size, surface coarseness, and variety of defects of the product surface, is an effective and direct sensory indicator for product quality inspection or production condition monitoring to a certain extent.

To achieve high robustness and computational efficiency of AOI, interdisciplinary knowledge on precision manufacturing and advanced image processing techniques is required in the novel system design. Problems and solutions are presented in the literature to increase the performance of AOI systems in various fields and forms. It is found that the correct diagnosis of some defects based on the image processing depends highly on the resolution and quality of the image acquisition, position of the piece, contrast and brightness of certain elements, lighting mode, etc.

Although the problem is very important for the production plants, the scientific literature does not abound with the presentation of the methods and systems that have been developed for this purpose. Some scientific papers, presented below, highlight the diversity of applications.

A triangular vision-based contouring error detection system and method is proposed in [2], realizing the three-dimensional error measurement of an arbitrary trajectory in conditions of a high feed rate and wide motion range. In [3], an approach for the automatic visual inspection that uses Multiple Instance Learning (MIL) for fault detection is proposed. The approach has been tested with data from three artificial benchmark datasets and three real-world industrial scenarios: inspection of artificial teeth, weld defect detection, and fishbone detection. The cornerstone for manufacturing improvement activities is intelligent equipment replacing human activities and that can be taught to replace human eyes, hands, and even brains; therefore, with each manufactured product, for quality diagnosis and for the completion of selection operation made by robots, basic data collection [4,5] must be prepared well and fast. According to a variety of hybrid image registration systems, [6] proposes an entire set of methods for achieving optimal hybrid pattern matching. This paper focused on using image server technology for high precision mechanical positioning.

A solution for the visual quality inspection in the context of the so-called Printing Industry 4.0 is provided in [7], where holes in the printing cylinder can occur during the process of producing gravure cylinders. To increase defect detection performance, this paper proposes a deep neural network-based soft sensor. The work is based on a high-resolution optical quality control camera and the technological readiness level has to be increased. An interesting study is presented in [8] focusing on the optical inspection of gear tooth surfaces using a charge-coupled device camera. The contour of the gear tooth surfaces is estimated and reconstructed. Another study, presented in [9], is associated with the battery industry in the context of a constantly growing market of electric vehicles and power storage. The laser welding is crucial in battery manufacturing, and consequently, the result quality check is important. Following applications based on convolutional neural networks for fault detection, recognition, and classification, this paper proposes an optimized visual geometry group model that contributed to a high rate of testing accuracy of 99.87%.

The solder joint quality inspection uses automated optical inspection. A significant study is presented in [10], associated with the welding defect inspection of the battery’s safety vent. As discussed in the study, a very important issue that seriously affects the image quality is the illumination environment.

The authors of [11] detail an improved 3D laser image scanning system to create an intelligent surface region of interest quantitative inspection strategy for a continuous casting production line. The laser image scanning system is based on binocular imaging and deep-learning techniques. The inspection method based on charge-coupled device scanning is mainly tied to the surface quality inspection for slab, strip, and billet products. A surface defect detection solution is presented in [12], where a spot scanning surface defect evaluation system is proposed. The study focuses on calibration and image reconstruction and the presented experiments show that a defect image with distortions within 1.8 pixels is reconstructed.

The production of steel parts, as rolling elements of roller bearings, requires quality assurance. In the context of reducing maintenance cost and increasing flexibility, the authors of [13] proposed a convolutional neural network for steel surface defect detection. The authors mainly approach the simulation of defects in order to reduce the disadvantages concerning neural network approaches that require labeled training data, but keeping high accuracy in defect detection.

Low-cost processing equipment for Printed Circuit Board (PCB) Assembly Automatic Optical Inspection is shown in [14]. Assembly processing defects, such as component missing, placement position inaccuracies, and other defects, could be detected using this equipment. The authors compare two common methods, the Canny Edge Detection and Sobel Edge Detection, with their proposed method, named Adaptive Gaussian Threshold. Image processing is done by a Raspberry Pi 3 Model B module with Ubuntu MateTM OS, with software applications written in OpenCV and Python. Additionally, a PCB optical inspection solution based on convolutional neural networks is provided in [15]. However, the situation encountered in these types of PCB faults detection research is not suitable for scenarios where the image quality is poor, captured from a higher distance and augmented by low-quality lenses, and where the physical equipment placement and operation do not allow higher image quality.

The current paper proposes an image-processing-based low-cost fault detection (IP-LC-FD) hardware–software solution for the End-of-Line (EoL) Electronic control units (ECU) in the automotive industry. It brings a new hardware architecture that interconnects several modules for parallel acquisition and processing of information; the results are collected quickly by means of a switch and are transmitted via Ethernet to the quality management system of the production line. The introduction of several acquisition and processing systems meant the elimination of telecentric lenses and the movement (translation/rotation) of the tested module, which leads to a massive reduction of costs. In the proposed constructive variant, the testing solution can be integrated on the production line and thus eliminates a machine and the related personnel for testing the ECU modules. From 150,000 euros to the costs of a similar machine at present, only ~3000 euros will be spent through the proposed solution. As for the processes above, quality control is very important in the manufacturing of automotive parts. In a brief description, an ECU consists of a PCB that is previously analyzed using Automatic Optical Inspection (AOI) and afterwards sent for pin insertion. Small and large pins are inserted according to specific layouts and structuring of parts. The products are then packed in enclosures and sent to the end of the production line for AOI and packing in boxes. The IP-LC-FD solution is conceived to perform AOI at the EoL, to identify defects at the pin, clips, and case level, and to be implemented on the current working production flow both as mechanical structure and as software structure to be included in the task workflow and traceability application.

The IP-LC-FD solution has to cope with the requirements of the real production facility, and it has to function in the production line. Therefore, the research had to be realized based on solid theoretical grounds that are suitable to be applied in practice short-term. Unlike many other studies in the literature, the study had to be developed rapidly from grounds to prototype, with an important milestone being the experimental model phase. The low-cost objective was a priority, and therefore, telecentric lenses, high-quality cameras, 3D scanning sensors, or high-resolution processing devices were not options. Besides the high price of some devices, the production line characteristics were not suitable for augmentation with some of the mentioned sensing structures, an important feature being that the solution should augment the current mechanical equipment in the production line, in order to keep the traceability intact. The 3D scanning sensors from [16] would fit the required enclosure, but the usable output of these devices in the context of the current AOI was lower than the currently proposed structure. This paper presents the proposed solution from hardware and software perspectives.

**Figure 1:**
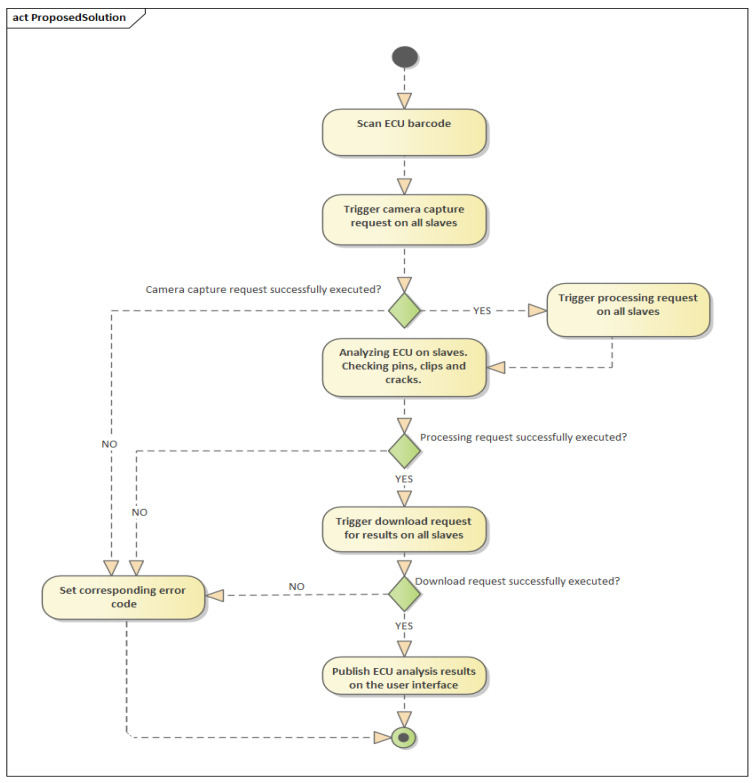
The abstract structure of informational flux in the image-processing-based low-cost fault detection experimental equipment.

According to Figure 1, the analysis process is started by scanning the barcode of the ECU, the master having connected via USB a laser scanner. When scanning a valid barcode (corresponding to the ECU to be analyzed) the master transmits an image capture request to all slaves on the network. Followed by the analysis request, the pins, clips, and cracks are checked on the newly captured image. The results calculated by the slaves reach the master following the transmitted download request and they are published on the graphical user interface. If the requests were not executed successfully, an error code is sent back to the master and it is displayed in the graphical interface.

The experimental model is first detailed, followed by the prototype description. The third section focuses on the obtained results, proving the efficiency of the solution, followed by a final discussion that concludes the study.

## 2. Materials and Methods

Due to the complexity of the project, the research followed a two steps approach. The first step consisted of the experimental model, presented in Section 2.1, taking the solution to TRL4. The second step, presented in Section 2.2, consisted of growing the solution to TRL7, the prototype level.

Research manuscripts reporting large datasets that are deposited in a publicly available database should specify where the data have been deposited and provide the relevant accession numbers. If the accession numbers have not yet been obtained at the time of submission, please state that they will be provided during review. They must be provided prior to publication.

Interventionary studies involving animals or humans, and other studies require ethical approval must list the authority that provided approval and the corresponding ethical approval code.

### 2.1. Experimental Model

The IP-LC-FD experimental model’s main target was to cope with the company’s fault detection rate requirements considering a major cost constraint. The target for the experimental model was to obtain at least a 95% fault detection rate and to obtain a processing time under 7 s. The cost had to be reduced as much as possible, primarily in the hardware part, followed by the software and equipment maintenance costs.

The main cost impacting hardware was the telecentric cameras. The central area of the object increases simultaneously with the cost of a telecentric camera. The first ECU to be analyzed using the experimental model was around 21 cm × 18 cm. The IP-LC-FD had to be placed at a certain level so that an operator could easily handle the analyzed ECU.

Radial distortion is a phenomenon specific to the image capturing phase which is caused by perspective (not all points from the captured image appear as seen from a perpendicular angle). Because of this, the pins appear as being offset from the central point of their corresponding slot. The issue was solved by placing the cameras in optimal positions and by creating a parameter for the origin point for each of the pins assuring the distortion is taken into consideration.

Therefore, the hardware architecture of the IP-LC-FD experimental model, presented in Figure 1, is centered around Raspberry Pi 3 boards and Pi cameras. One board and camera ensemble cost more than 100 times less than one telecentric camera, but the Pi camera will always present perspective over the board, which is a major disadvantage. Additionally, the graphic processing ability of a Raspberry Pi is much less than a PC with a decent video card.

**Figure 2:**
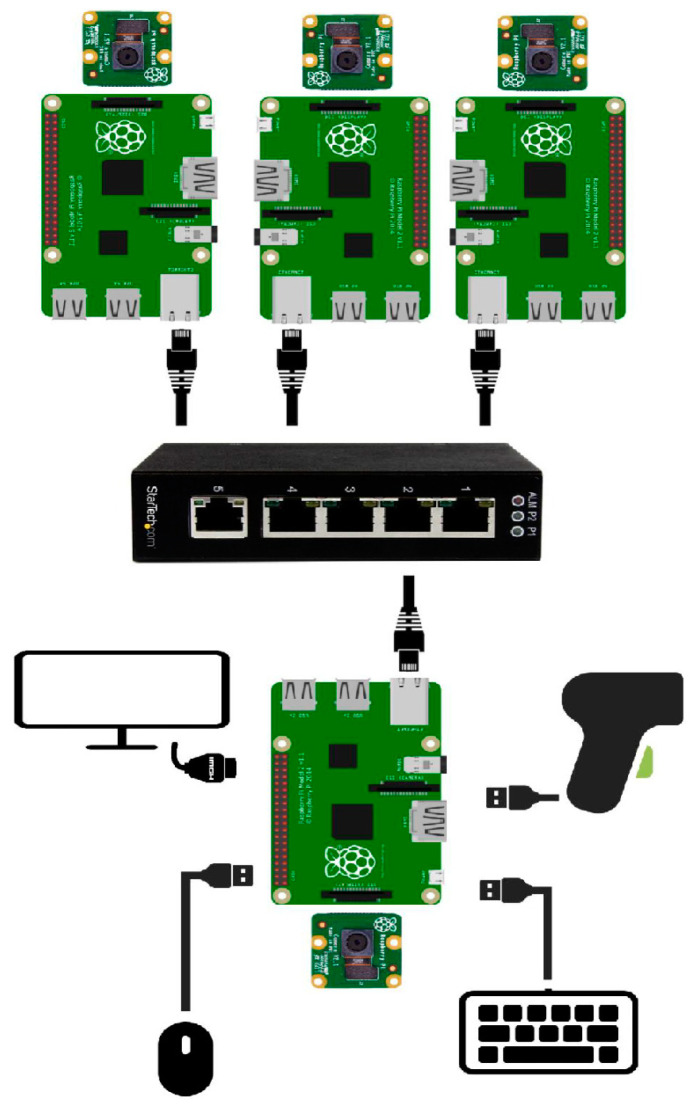
The hardware architecture of the IP-LC-FD experimental model.

The hardware architecture presented in Figure 2 consists of:

- Four Raspberry Pi 3 boards. One board is the master and the other 3 boards are slaves.
- Four V2 Pi cameras. One Pi camera is attached to each Raspberry Pi and placed inside an enclosure. Each Pi camera communicates with the Raspberry Pi through the Inter-Integrated Circuits interface (I^2^C). On each enclosure, a 2× telephoto lens is mounted. The lenses are placed at 31 cm above the superior area of the ECU, with an error range of ± 0.5 cm on the vertical axis.

**Figure 3:**
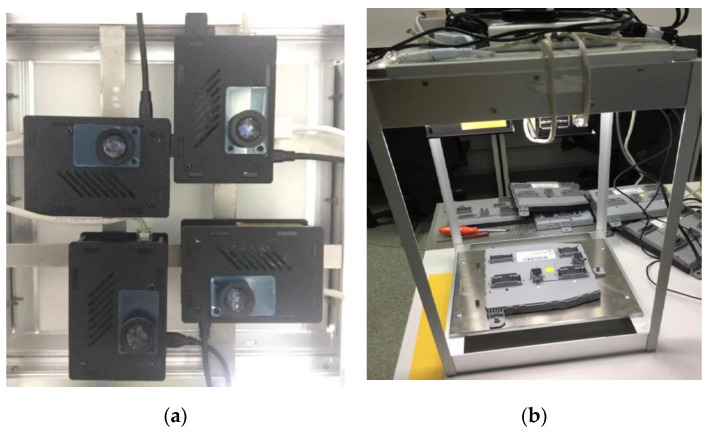
The experimental model hardware of the IP-LC-FD. (**a**) The disposal of the four Raspberry Pi–Pi camera ensembles. (**b**) The final experimental model stand.

The final positioning of the enclosures in the experimental model is presented in Figure 3a. The final structure of the experimental model presented in Figure 3b includes a uniform lighting structure placed beside the enclosures.

- One Ethernet switch with 5 ports without management. Four ports are used for internal communication between the master and the slaves (the IPs are manually set for each device) and 1 port was planned for external communication inside the factory network.
- One Barcode scanner. The Barcode scanner is a usual, linear scanner, transmitting the barcode through the RS232 serial interface, the USB port. Although as an alternative, a barcode reader software module was implemented by the authors, the external scanner and an interfacing and wrapping software module were preferred to be able to further align the cameras and to further reduce the processing time.

Each ECU module is divided from the image processing point-of-view into 4 zones/areas. Each area is photographed by a camera connected to a Raspberry module. The ECU board dimensions are significant and, therefore, the 4 areas were necessary in order to handle properly the perspective and the shadows in the image for correct processing. The switch, introduced in the hardware architecture, allows the parallel processing of the areas of the image corresponding to each assembly formed by the camera and the Raspberry module. Therefore, the processing speed is increased by this parallel processing and also by using threads in each Raspberry Pi software.

Regarding the software and equipment maintenance costs, the flexibility, adaptability, and modularity were the main requirements to: easily replace modules; be expandable to function with different types of ECUs; be able to learn new configuration for an existing type of ECU; configure from the Graphical User Interface (GUI) parameters as lighting, area, search position, etc.; function without moving parts and, therefore, reduce supplementary equipment maintenance.

In order to work with less expensive and complex hardware, the researched image processing fault detection solution had to overcome the drawbacks and provide the same or even better solution.

**Figure 4:**
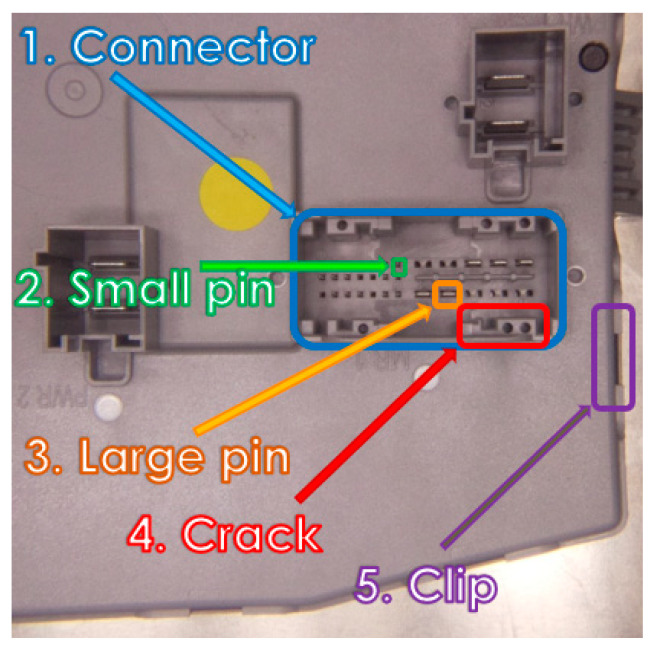
Some analyzed components on an Electronic Control Unit (ECU).

The experimental model proposes a low-cost hardware–software solution, based on image processing, that detects faults (e.g., pins, connectors, clips) on specific ECU boards at the production EoL. For a better understanding of an ECU structure and the requirements, Figure 4 presents examples of large and small pins, connectors, clips, and cracks on an ECU.

The main requirements and consequently the implemented functions were:

- Detection of crooked pins;
- Detection of missing pins;
- Third bullet;
- Detection of extra pins;
- Detection of wrong clips disposal or damaged clips;
- Detection of cracks on boards;
- Reporting of the whole process;
- Collecting and marking faults, data aggregation on the master equipment, creating logs, user and board selection, debug procedure, etc. within the graphical user interface;
- Managing existing boards configuration;
- Learning new board configuration;
- Reading barcodes;
- Data exchange between the four microcomputers and communication with the higher-level traceability application.

The used software environments were Python, PyQT, and OpenCV on the Raspbian operating system. From an architectural point-of-view, there are two types of actors: master and slave.

The master has the authority over each slave in the sense that it dictates what to do (e.g., image processing, image capture, prepare the processed image for download). Each slave serves only one client, the master.

The entire communication between the actors is realized through Ethernet. Each slave type node has implemented a server. The protocol for the data exchange between the nodes is Remote Procedure Call (RPC), a request–answer type of communication used to synchronize processes. An RPC is initialized by a client that makes a request to the known server and calls a method, registered on the server. These methods may also receive parameters from the client. These parameters are either XML (standardized data) types of JSON (data dictionary with keys and values) types.

The master coordinates all the slaves in the local network through Remote Procedure Call (RPC) requests. Through this communication protocol, the master can call the execution of a procedure directly on the slaves and receive a response back. The master decides which tasks and in what order are executed for each slave in the network.

The purpose of a slave is to execute commands received from the master and to transmit the execution result back. A slave is a loop application that waits for requests from the master to process them.

During the execution of the processing request, the master is on hold until all slaves finalize the execution of the request. Each result calculated by slaves is composed of analysis status and error code. The error code received from slaves is nonzero only if there were problems executing the requests. RPC may be considered as a type of Inter-process Communication (IPC), mentioning that these processes are situated on other computational systems. In the case of functional changes of the system (adding another module for processing, connecting other cameras for image capturing), the server and the RPC type requests will remain intact. This architecture is illustrated in Figure 4 and the main modules are detailed in Section 2.1.3.

**Figure 5:**
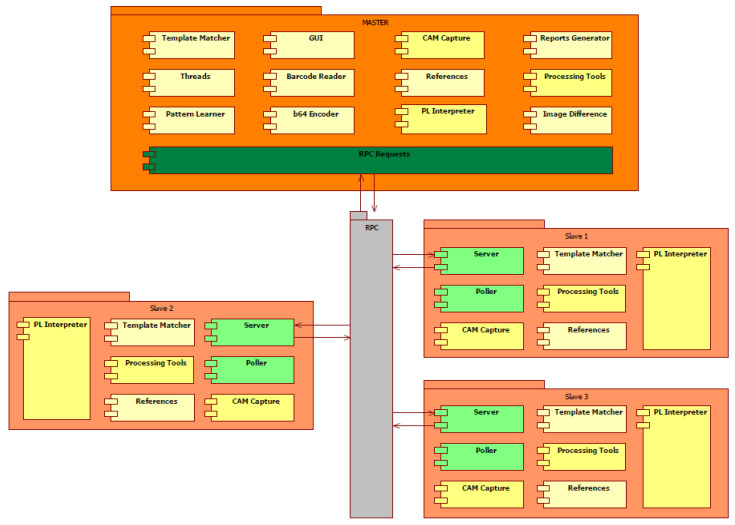
Some analyzed components on an ECU.

Figure 5 presents the communication between the nodes and the implemented modules. The RPC requests at the master node are represented with dark green, communicating bidirectionally with the server of each slave node. Additionally, the Poller module has an important role in the communication part because it manages all the requests coming from the Server. On the master side, all the connectivity errors are caught using the protocol that offers large Application Programmable Interfaces (APIs) that handle different types of errors.

The color code in Figure 5 presents:

- The beige-white blocks are specialized and specific modules for certain types of operations. These are unique modules for each node, not having similar code parts with other nodes.
- The yellow blocks are distributed over various nodes and have similar functionalities.
- The green blocks (light or dark) are representing modules that are handling the communication between the nodes, specifically requests sending and analyzing, prioritizing, and interpreting. Additionally, these modules assure the keep-alive connection maintenance and command task executions for each node.

#### 2.1.1. Master Node

**Figure 6:**
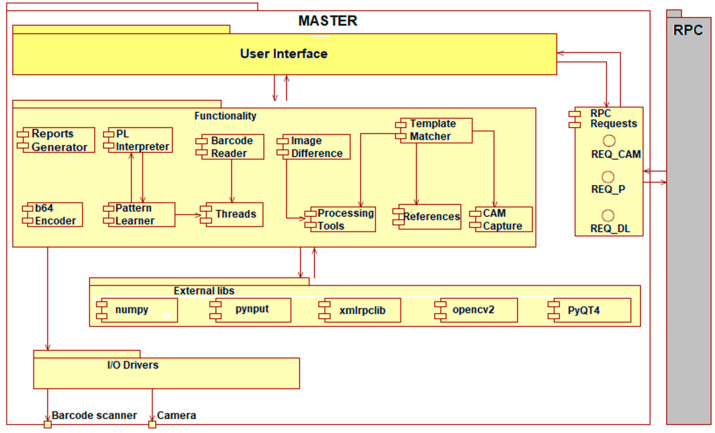
Software architecture of the Master node.

The master node represents the main element in the entire structure, containing command and control functionalities and being the only node that can communicate with the other nodes. From the software point-of-view the master node contains the following components (see Figure 6):

- I/O Drivers: the component that handles the communication between the microprocessor and the peripherals (camera, barcode reader).
- Functionality: the component contains the functional modules of the master node, assuring the image processing, data interpretation and processing, image capturing from the peripheral, barcode scan interpreting, etc. The functional component is dependent on the Drivers component and situated on a superior level towards I/O. Any superior component regarding the Functionality cannot communicate directly with the drivers. Besides the Python language, there are some external modules dependencies:
  ○ numpy: library used for n-dimensional matrix calculus, higher-level functions, linear algebra, generical multidimensional data.
  ○ pynput: library to control all input devices.
  ○ xmlrpclib: Ethernet communication protocol.
  ○ opencv2: used for image processing.
  ○ PyQt4: library used for user interaction, creation of interfaces.
- RPC requests are the component that handles the communication with the slaves. These requests are ordered by the master and executed by the slaves. Through the Local Area Network (LAN), the master transmits the following requests:
  ○ REQ_CAM: request for capturing an image.
  ○ REQ_P: request for ECU analysis (pin detection, clip detection, crack detection).
  ○ REQ_DL: request for downloading the final results after the analysis of slaves. The results downloaded on the master are used to display them in the user interface and add them to the final analysis report.

It is mandatory that the order of execution of the requests is REQ_CAM->REQ_P->REQ_DL and it is coordinated by the master. If any of the slaves fail to execute the requests, the analysis process is canceled and flagged with an execution error.

- UI (User Interface): All technical details regarding implementation and all procedures to obtain outputs are situated in the Functionality component. From the UI, the user sends commands that trigger the Functionality component. All these commands are covering all the components from the master node, and the received answers are sent back to the component on the highest level of the architecture. Additionally, UI launches in parallel the requests to the other nodes within the system.

**Figure 7:**
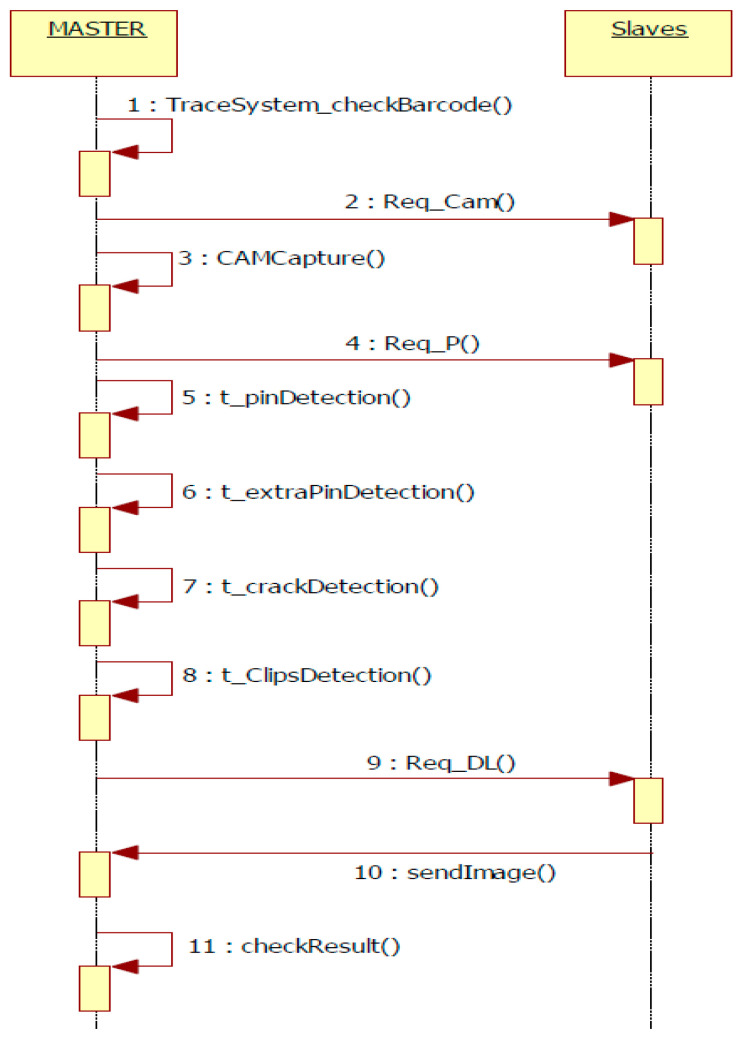
Processing flowchart.

The IP-LC-FD application contains two types of processing: debug processing when the barcode is not read and it is NULL, and therefore, the traceability system does not confirm the board as being on the stand and ignores it; normal processing: the barcode is transmitted to the traceability system and the detection is registered, waiting for a final status of the process (PASS/FAIL). The requests from the master node to the slaves are executed in the following order (see Figure 7): request for slave image capture (REQ_CAM), request for processing (REQ_P), and request for download (REQ_DL). The same order of execution is on the master node.

#### 2.1.2. Slave Node

**Figure 8:**
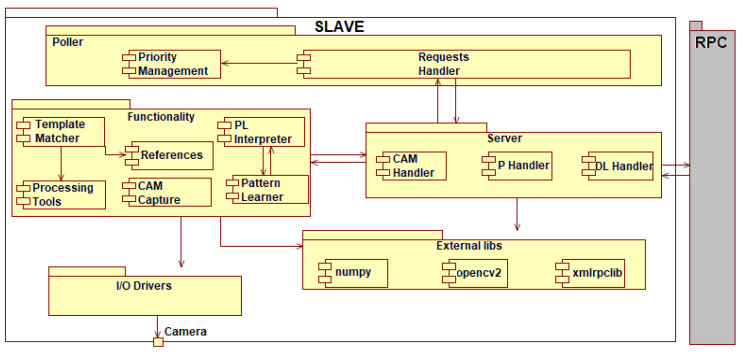
Software architecture of the Slave node.

**Figure 9:**
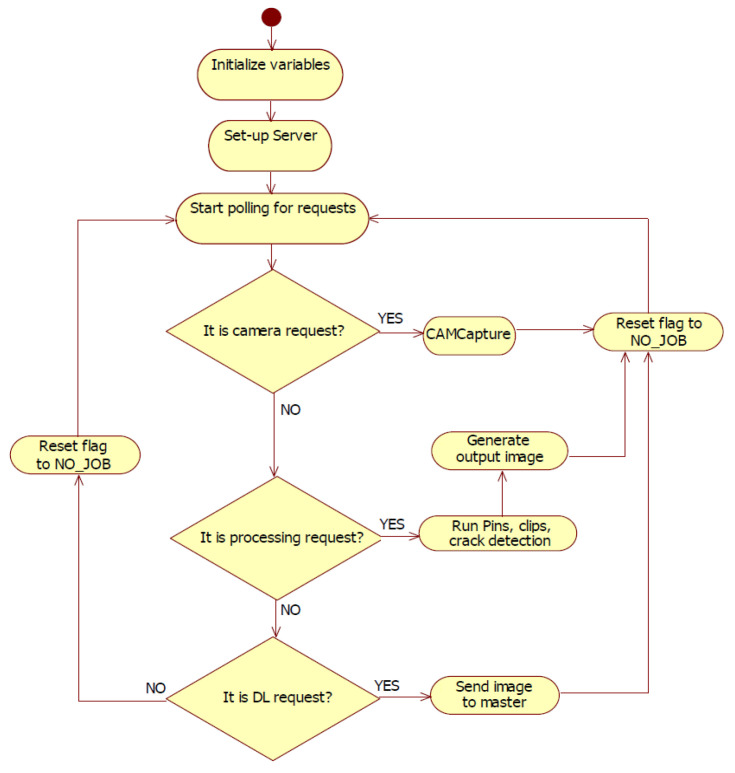
Slave node diagram of activities.

Considering the software architecture, the slave node is organized on components according to the provided functionalities and the interaction with other modules (see Figure 8 and Figure 9). The architecture base contains the I/O Drivers (input/output drivers) that command the peripherals and the external libraries that represent the core for the other components. It contains the .dll files (Dynamic Libraries) for xmlrpclib, opencv2, and numpy. Considering the low-level elements, the two major components that depend on drivers and external libraries are the Functionality and Server components.

The Functionality component contains all modules that are necessary for image processing, data interpretation coming from the Ethernet, and references. The Functionality component is accessible only through the Server component.

The Server component interprets the requests coming from the master node and contains three handlers:

- CAM Handler: Accesses the CAMCapture module from the Functionality component and captures the image.
- P Handler: Accesses the processing modules (Template Matcher, Processing Tools) and realizes the pin, clip, and crack detection. All these are marked on the captured image.
- DL Handler: Transforms the processes image into binary data and realizes the data transfer towards the master node.

The main component of the slave node is the Poller. The Poller allows the indirect access form for each module and establishes the workflow for the entire node. The Poller waits for master requests and adds them in a queue according to the priority of each request (see Figure 9). The priority is established considering the current request’s dependencies. Therefore, a processing type request cannot be executed without an image. If there is also an image capture previous request, the processing is launched. If no Priority Management module is found for the Poller, the processing request automatically launches the image capture request.

#### 2.1.3. Main Modules

**Figure 10:**
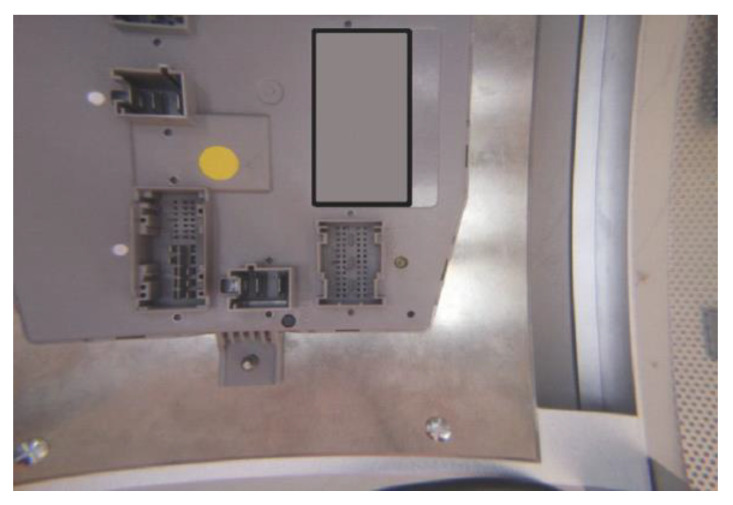
Initial image taken over by a camera.

The system is modularly designed so that each module has an individual well-defined and targeted scope, with simple links between modules. Some modules contain configuration files for system calibration. The software development is divided into 15 main modules, each containing smaller functions, and the subsequent detailing level is brief considering both the large system implementation and the confidentiality issues regarding the final beneficiary of the solution. The modules are:

- CAMCapture: Used to take over the saved image in a file by the primitives from the PiCamera library. An important aspect is that the camera resolution is set at 2592 × 1944 pixels. Regarding hardware considerations (meaning that the image is distorted through the lens), the useful surface of the image is much smaller than the initial picture that can be observed in Figure 10.

In Figure 10 and next figures, the gray rectangle was introduced to avoid advertising the products under test.

**Figure 11:**
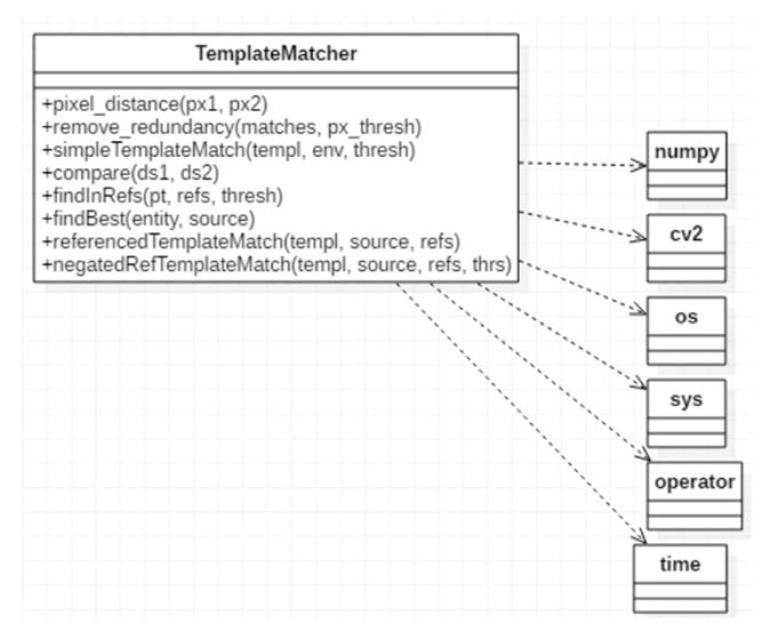
Class diagram of the Template Matcher module.

- Template Matcher: The functional core of the application, containing basic methods for processing the input image. The Template Matcher class diagram is pictured in Figure 11 and uses primitives from the OpenCV library, as well as low-level; processing techniques, working directly on the color values of the pixels.

**Figure 12:**
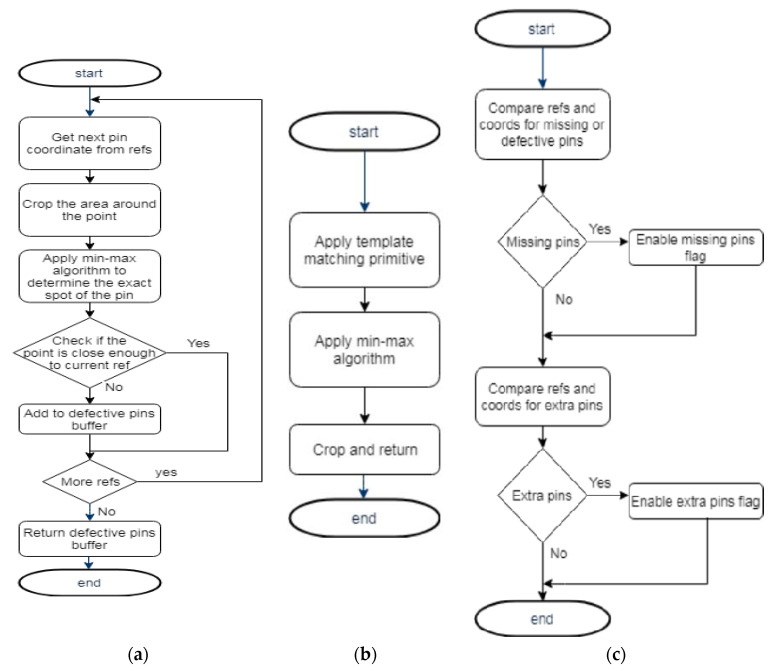
Flowchart of the function (**a**) referencedTemplateMatch; (**b**) findBest; (**c**) compare.

Arguably, the most complex method of the module is the “referencedTemplateMatch” that handles the detection of the smallest pins installed on the ECU. This method has three input parameters: *templ* (template), *connector,* and *refs* (references). The *templ* parameter is a template image from the file structure of the project, used to identify matching zones within the source image. The source image is given as the *connector* parameter, and actually represents the crop of a specific connector from the ECU. The *refs* parameter is a list of coordinates, received from a stored set, that is previously processed by a Pattern Learning module that chooses the selected coordinates from the user interface and returns them for processing. The list of coordinates is used to optimize the pins detection process, considering the prerequisite that the pins will always be found more or less in the same positions altered by a maximal specific area. The main steps to detect the actual position of the pins and to decide if a pin is inside the correct threshold zone in order to be stated as valid, are presented in Figure 12a.

If the pin is not valid, its coordinate will be added to a list of faults that will be later returned to higher levels of the application and will be used to mark the faulted pins with a red square on the user interface.

A connector from the ECU is found also through a method from the current module, called “findBest”. Its flowchart is presented in Figure 12b.

The last presented method of the module is the *compare* function and realizes the extension of the so-called correct set of pin coordinates provided by the Pattern Learner module. The *compare* method (the associated flowchart is presented in Figure 12 analyzes first if the detection of pins results according to the received set of coordinates). If no pin is detected in a spot that it should be, it is considered as a missing pin. Additionally, the *compare* method analyzes the whole set of coordinates associated with the specified ECU, and searches if an extra pin is detected where it should not be. The extra pins detection is somehow in an opposite logic as the basic pin detection.

- Threads: The module contains all types of threads used in the application. During runtime processing routine, multiple resources are permanently checked so that the execution time will not be significantly affected. One of the resources is the available memory to store the reports. Periodically, the location of the reports is diagnosed and a byte dimension calculus is realized, the result being shown as a status bar filled in percentage (0–100%) on the user interface.
- Pattern Learner: The IP-LC-FD application is foreseen to analyze more types of ECU boards as long as they have the same mechanical structure. During the first phase of researching the experimental model, the provided boards had the same mechanical structure, but the configuration of the pins within the mechanical structure determined various functional characteristics having different corresponding circuits in the background. In industry, it is a common procedure to use the same mechanical structure for a large set of ECU board types in order to reduce the production costs. Therefore, the Pattern Learner module was created in order to validate various types of boards, and it exposes all connectors with the pins’ configuration to the user in a window. The configuration of pins can be adjusted (add/remove pins) within the interface, and the configuration can be initialized, created, and viewed in the window.

In the experimental model, the Pattern Learner implementation needed 31 vertical and horizontal layouts to be able to position pins and connectors exactly as in reality. All connectors are positioned on 3 main layout groups (superior, central, inferior), each belonging to the root layout. The advantage of the structuring is that each connector will remain in the same format as the processed board regardless of the monitor resolution. The layouts are not allowing the interface distortion, each element within the interface is rescaled according to the window dimension.

**Figure 13:**
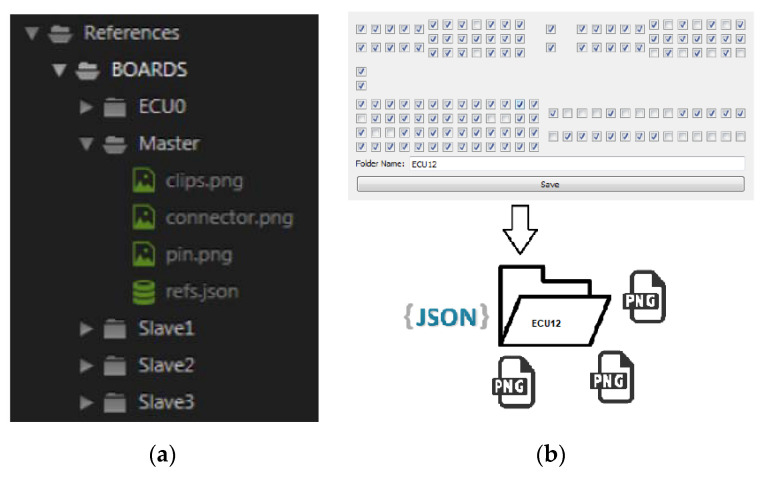
References (**a**) file structure; (**b**) saving procedure.

- Graphical User Interface (GUI): The GUI module contains all elements on the superior layer of the project, the user interface. These elements (buttons, textboxes, console, windows, combo boxes) are allowing the user to interact and configure the entire system.
- Barcode Reader: The software module is found only on the master mode and it is compatible with many types of barcode readers. The module launches the execution start signal for the application when the system is in operator mode.
- Base64: The module is used to encode the images into arrays of char to ease the network transport. For example, if the images are transferred through the network as pure binary data, compatibility issues may occur between operating systems. Additionally, some protocols may interpret binary data as special characters. All binary data are encoded as ASCII text.
- References: Creates references for a certain type of ECU. The references are used for image processing, contain data regarding elements that must be processed on the board and basic captures of elements to be identified. Using these references, the processing modules decide if some parts of the board are deteriorated. The references’ file structure for a board is exemplified in Figure 13a. The references are saved and named within a folder, containing a template as a .png file and a .json file with the pins’ layout on the boards (see Figure 13b).
- PL Interpreter: The PL (Pattern Learner) Interpreter module takes over and translates the Boolean values from the GUI and returns the coordinates for the pins. The module has two types of filters applied to the pins and connectors from the interface. All marked pins on the interface are verified and validated (valid position within the hole, not broken, not bent, etc.) and all unmarked pins are verified to determine if there are no extra pins on the board.

**Figure 14:**
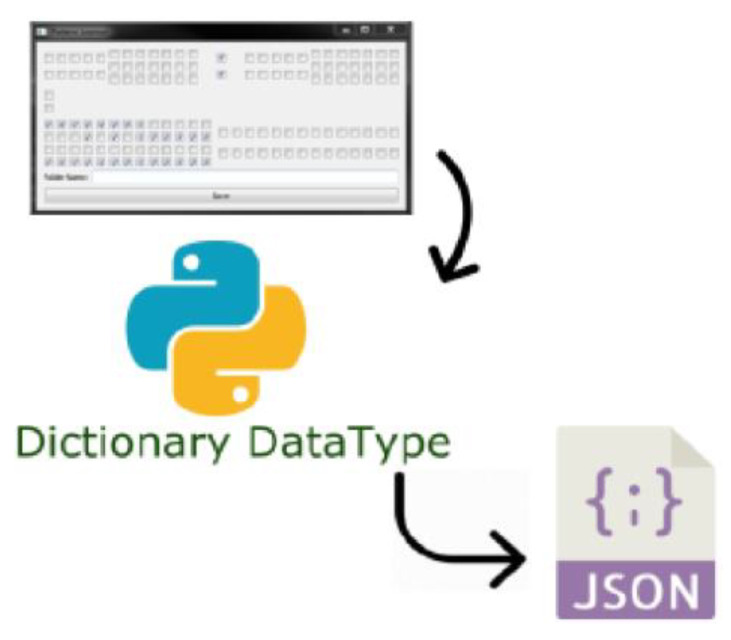
Data saving model.

Each pin from each connector is checked in a certain order, resulting in a dictionary data type that contains the primary key, the name of the connector, and as value, a list of Boolean values. The dictionary is saved in a json file, as a resource for the reference (see Figure 14).

**Figure 15:**
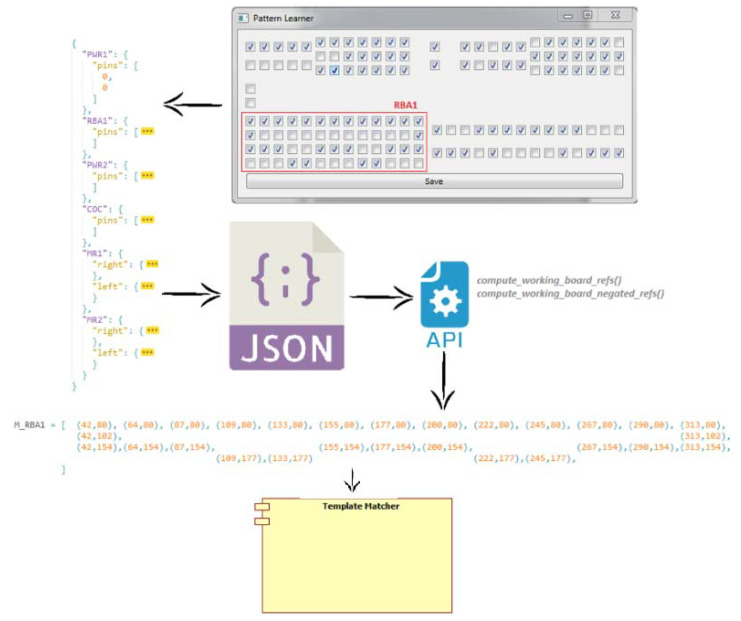
Coordinates conversion process.

Starting from high-level objects, QCheckBox, the value of each object is extracted and grouped in the dictionary. The obtained file is transmitted by the master node to the slaves, is loaded in the RAM, and further on two functions are applying the corresponding filters to extract the pin coordinates (see Figure 15). These coordinates are stored as lists of tuples and used as inputs for pin detection methods within the TemplateMatcher module.

**Figure 16:**
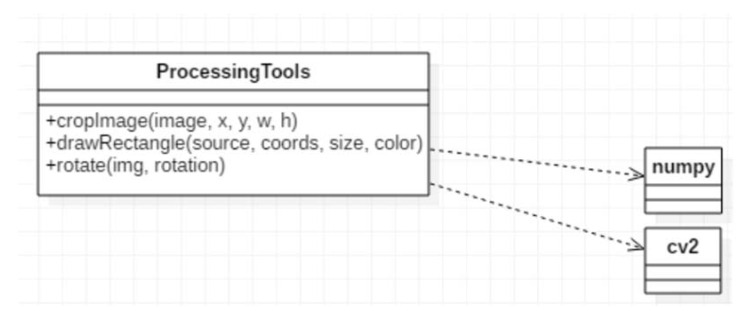
Class diagram of the Processing Tools module.

- Reports Generator: The module generates reports after finalizing the processing. All information regarding the analysis is stored within a folder.
- Processing Tools: The module contains methods designed to assist other processing modules to accomplish the detection. The class diagram of the module is presented in Figure 16. The methods are impacting the main functionalities indirectly, but their separation is important from the resources point-of-view. The impact of the methods in the overall detection process is significant. For example, rotation procedures are applied permanently, either using straight angles to adapt each camera position, or in small angles (within 10°) for correlating and adapting the mechanical degrees of freedom in placing the boards for image processing. If the image is not well-rotated, the cropping procedure may cause the loss of important data. Additionally, cropping is essential for detection, but also to largely reduce the execution time.

The cropping made before image processing is an optimization method. From the captured image is cropped an image delimited by a set of given parameters (as coordinates). In the cropped image a template matching algorithm from the OpenCV library is applied with the purpose of detecting the connector. This algorithm is not invariant to scale and rotation as opposed to other algorithms such as Scale Invariant Feature Transform which would be more costly from the perspective of processing power. For this reason, the rotation of the captured image caused by external factors (such as misplacement of the camera) can cause data loss.

**Figure 17:**
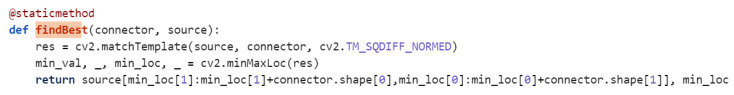
Best match connector search function using the matchTemplate primitive.

The function from Figure 17 has the purpose of searching the best match of the connector using the matchTemplate primitive from the OpenCV library. The function takes as parameters the captured image and a reference image of the connector and returns as a result of a matrix with the best match of the connector cropped from the captured image.

- Image Difference: The module is used for cracks detection or other nonconformities on the boards. The module marks with a red contoured rectangle the problematic area to be easily observed by the operator.
- RPC Requests: The module was previously described.
- Server: Each slave contains a Server module. The modules run on separate threads and serve all requests on a port. The module is implemented using the RPC protocol and assures the communication between the master and the slaves. A set of methods are registered and can be called, all other methods or request types are ignored.
- Poller: The module was previously described.

### 2.2. Prototype

The starting point of the current paragraph is represented by the experimental model of the IP-LC-FD system. The activities to reach the technological readiness level associated with a prototype were to adapt, improve, test, and validate the software from the experimental model within a new mechanical–hardware structure. The new mechanical–hardware structure was necessary because of the requirements in the production line and to achieve the necessary performance for more basic types of ECUs.

The prototype was conceived and implemented, and the solution was integrated, tested, and validated in the production line. Some results are detailed below:

- Starting from the experimental model (consisting of 4 compact modules, each containing a Raspberry Pi and a camera), the solution was modified within the prototype in order to fit the high number of different ECUs from the production line and to reach the test indicators. A prototype solution was conceived and implemented, based on 6 Raspberry Pis and 6 cameras, this time physically separated, and improving the quality of the inspection for the connectors with perspective issues. Having separated cameras, the prototype allows besides higher possibilities to eliminate the perspective, a higher luminosity on the ECUs analyzed surface.

**Figure 18:**
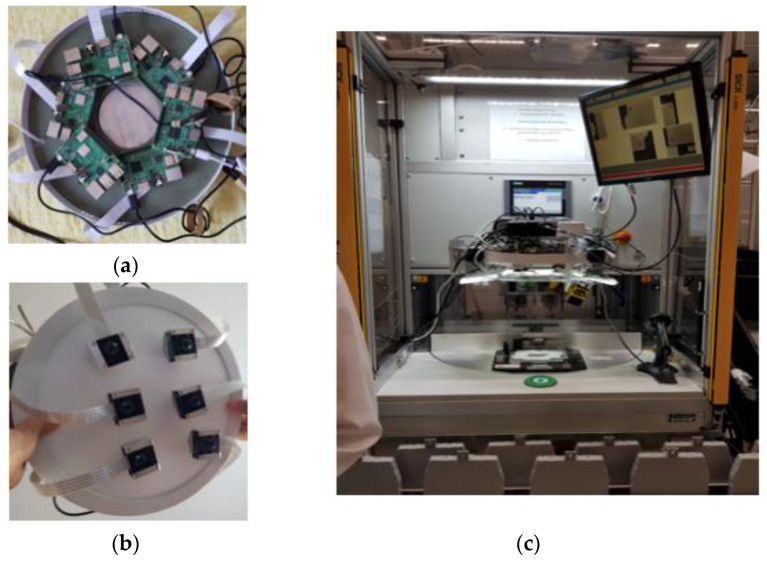
First phase of the IP-LC-FD prototype (**a**) upper side view over the installed Raspberry Pis, (**b**) lower side view over the cameras that are magnetically attached to the illuminating surface, (**c**) the prototype installed on the operational machine.

The first mechanical–hardware prototype was a circular mechanical surface and the cameras were magnetically attached to the surface. The structure is depicted in Figure 18.

**Figure 19:**
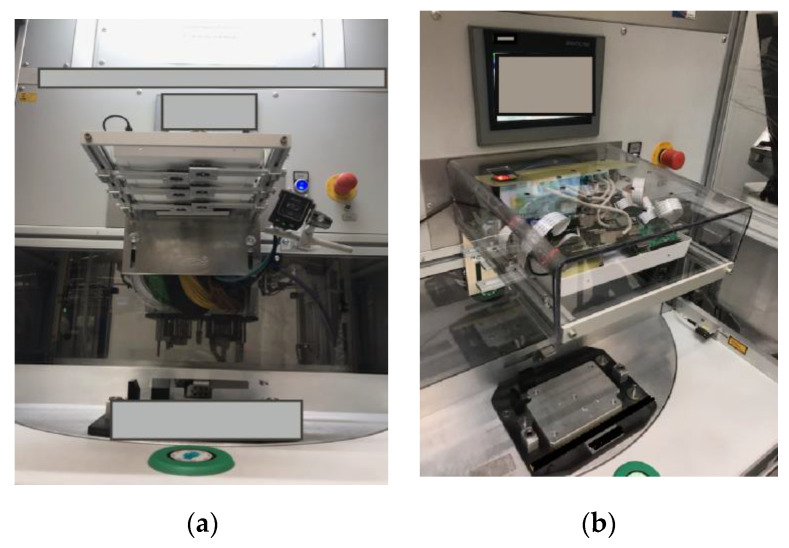
Final IP-LC-FD prototype (**a**) lower side view over the cameras installed on a specially created adjustable mechanical structure, (**b**) upper side view over the prototype installed on the operational machine.

During the first round of experiments, the position of the cameras was many times accidentally changed by the operators, disturbing the whole detection process, sometimes requiring the change of references (recalibration) of the moved camera. Therefore, the final rectangular mechanical–hardware prototype is illustrated in Figure 19, where the camera’s position is easily adjustable and, in the meantime, increases their stability. On the right-side image, a protection shield was installed.

**Figure 20:**
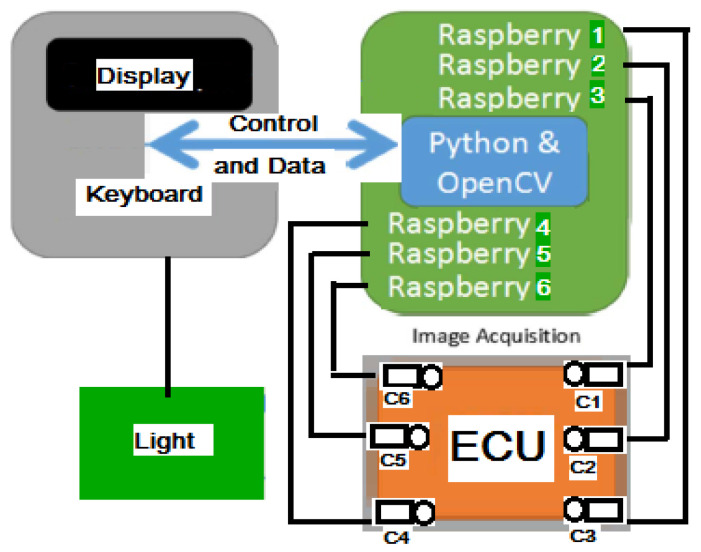
Schematic view of the IP-LC-FD prototype.

- The capability to vehiculate data was extended to be between 1 master and 5 slaves, including from request/response transmissions toward data aggregation, concluding procedures, and reporting. The schematic view of the prototype is shown in Figure 20, each Raspberry Pi analyzing a zone from to the board, captured by the corresponding camera.
- A generic platform was created for n processing modules. Therefore now, the prototype contains only two branches: 1 master branch and 1 slave branch, and the software from the slaves is generic for any Raspberry Pi slave in the scheme. This aspect brings many advantages during maintenance because any module can be easily replaced and configured.
- The prototype solution is extended to function for the main classes of ECUs from the production line. The extension required a new concept for the software module. This was necessary because of the differences between the hardware-mechanical structuring of the main classes and the necessity of the software to adapt to all cases.
- Modules were conceived and developed to include layouts from all main classes of boards and all the particular sets of boards inside the main classes.
- The pin search module was optimized through additional cropping to reduce the search area and the processing times.
- New detection modules were researched and implemented. The new detection modules were based on “islands” identification. The identified islands were then either separated or grouped, for more accurate conclusions.
- A new method was researched and developed to establish a dynamic illumination threshold associated with each pin. The light is crucial, and the prototype aims for accurate detection in the open working environment. The experiments showed that if the structure is not placed in a closed environment, light and shadow differences will occur. The pins and the pinholes are small; therefore, the different illumination thresholds were necessary.
- The layout saving, storing, and loading was optimized due to the high number of layouts in the production line.
- Detection task request optimizing was researched and implemented mainly in the sense that the master equipment extracts and sends a list of the connectors to analyze for each slave equipment. This procedure eliminates the fixed slaves, each being able to be replaced without application issues.
- The processing time was significantly reduced by eliminating the necessity of connector rotation for the slave’s software.
- A new module was implemented for new layout learning, placed only on the master equipment. This way no ssh/vnc connection is necessary for each slave.
- A new offset (search area) separation was researched and implemented so that each pin has now its own offset. Additionally, a new module was created that establishes the filling factor for the offset for each pin.
- The prototype is able to apply all the changes in configuration from the graphical user interface.
- The prototype functions in complete correlation with the traceability software within the company.

## 3. Results

Both the experimental model and the prototype were tested rigorously and validated according to their specific technological readiness level.

For the experimental model, the testing was realized in the laboratory, using the developed IP-LC-FD stand. Around 40 real boards were used from one class of ECUs. Various ECUs had different pin layouts and were, therefore, able to test various board configurations. In order to analyze as many situations as possible, some ECUs were manually modified and even destroyed in the following manner: pins were bent, broken, cracks were induced on the boards’ surface, clips had been broken. Additionally, the stand itself was analyzed to achieve the best performance in the context of keeping the mechanical requirements from the production line. The conclusions were that the 30 cm × 30 cm diffuse light surface was necessary to be placed at the superior level of the stand, and the Raspberry Pi and camera enclosures to be installed at approximately 5 cm below the light surface. The ECUs were finally placed at 30.5 cm under the enclosures.

The prototype testing was realized over three months, in the production line. The prototype was tested on more than 1000 boards. The tests were carried out on four ECU classes, each of the classes having many different pin configurations.

**Figure 21:**
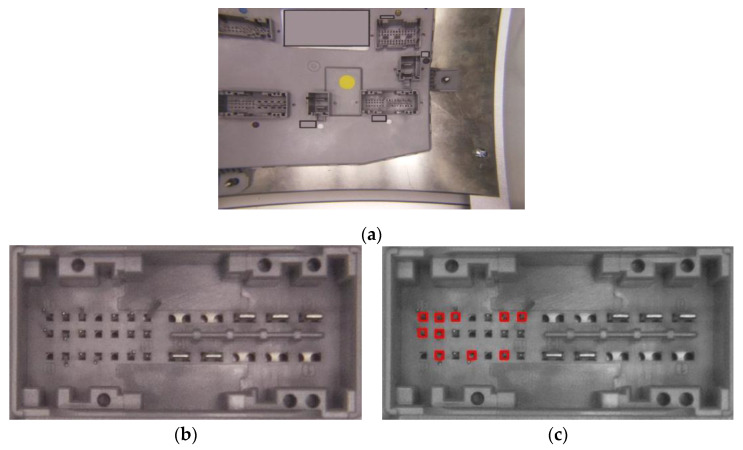
Results obtained with the IP-LC-FD system: (**a**) the source image taken by on slave; (**b**) the identified and extracted connector from the source image; (**c**) the faulted pins detection resulted after image processing.

The first scenario presents the fault detection mechanism for bent pins in a connector, analyzed by one of the processing equipment. Figure 21a shows the source image captured by the processing device. From the source image, the algorithm detects and crops the analyzed connector. The resulting image is presented in Figure 21b. Then, the algorithm detects the pinholes and the position of the pins in the holes. Figure 21c depicts the results of the fault detection algorithm, where the bent pins are marked with a red square, respectively, the correctly placed pins are not marked.

**Figure 22:**
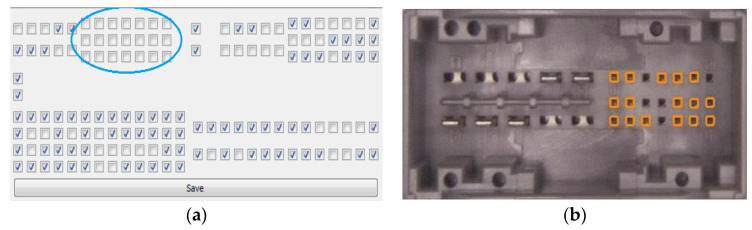
Results obtained with the IP-LC-FD system: (**a**) the unmarked pins in the Pattern Learner module for a connector; (**b**) the detected extra pins in the specified connector.

The second scenario presents extra pins detection. For the detection of extra pins, in the Pattern Learner Interpreter, all pins associated with a connector are unchecked (see Figure 22a). In this situation, the algorithm should detect all pins that are present in the unmarked positions in the corresponding connector, respectively, all the empty holes should be left unmarked. The results of the extra pins detection algorithm are presented in Figure 22b, all the detected pins being marked with orange.

In the three positions in Table 1, the detection with only 98% is due to the different gloss/oxidation of the surface/top of the pins, but also of the free place (pad), in which no pins are mounted. The freedom of movement of the ECU module of ±0.5 mm in the test system is another factor causing errors.

**Figure 23:**
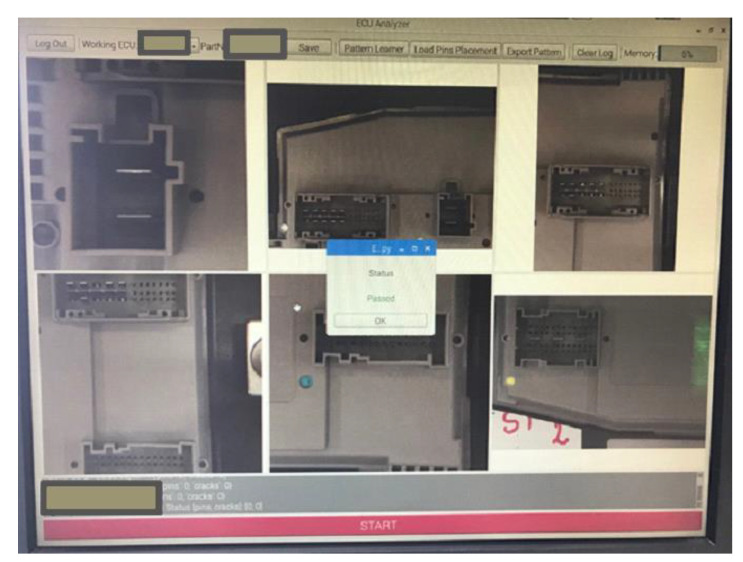
An overview of the user interface and a test result for no detected faults.

Table 1 presents other results of the testing procedure and Figure 23 presents a view of the user interface and a test where an ECU has no faults and the board is declared Passed.

**Figure 24:**
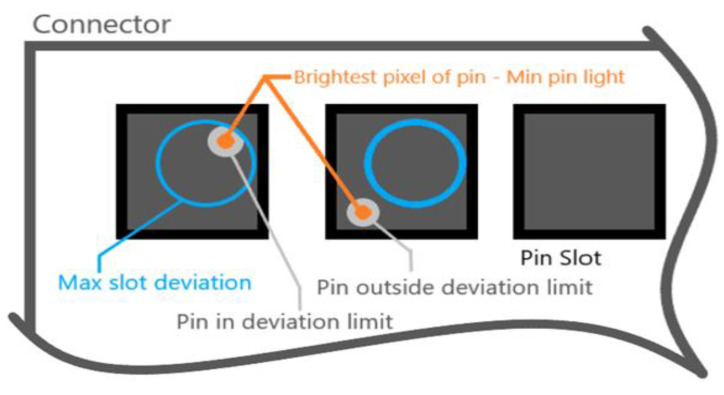
Some pins parameters.

A few of the parameters are presented in Figure 24.

- Max slot deviation is defined by a radius in pixels around the origin of the slot (which is also given as a parameter) that defines the maximum deviation a pin is allowed to have;
- Min pin light is the minimum intensity of brightness on a scale of (0–255) the brightest pixel of the pin is required to have to be considered a valid pin and not a background element.

These are the most important parameters (among many others) that determine the validity of a pin in a given position.

## 4. Discussion and Conclusions

The proposed low-cost solution, which is currently at the level of technological prototype training (TRL), is designed for the Automatic Optical Inspection (AOI) of electronic control units (ECUs) in the automotive industry. It brings a new hardware architecture that interconnects several modules for parallel acquisition and processing of information; the results are collected quickly by means of a switch and are transmitted via Ethernet to the quality management system of the production line. The introduction of several acquisition and processing systems meant the elimination of telecentric lenses and the movement (translation/rotation) of the tested module, which leads to a massive reduction of costs. In the proposed constructive variant, the testing solution can be integrated on the production line and thus eliminates a machine and the related personnel for testing the ECU modules. From 150,000 euros to the costs of a similar machine at present, only ~3000 euros will be spent through the proposed solution.

The IP-LC-FD system is able to identify the components of an ECU and it accomplishes successfully the fault detection in the context of a low-cost infrastructure.

The pins detection implied the creation of many algorithms that were tested and the one with the best performance was selected. The best performance was indicated by the smallest error rate, but also the processing speed was a requirement in the production line. The processing speed and the type of chosen algorithms itself was a challenge because of the used low-cost hardware (both cameras, lenses, and microcomputers). Therefore, the processing speed reduction was necessary for the selected algorithm optimization strategies. The processing speed was reduced by using threads, by proper cropping and rotations, by reducing the redundant calculus that was implied by using OpenCV library primitives. When expanding the entire structure for the prototype, the processing charge was also reduced for some slaves.

The production line requirements of positioning the ECUs implied the flexibility of the algorithm. The boards are not placed in a fixed position for testing. Therefore, the fault detection algorithm for the small pins in the experimental model was conceived so that it has a flexibility for vertical translations of around 0.7 cm, and for horizontal translations of around 0.1 cm.

The algorithm used for the large pins and clips is even more flexible, the translation degree being controller through the configuration files parameters. The same situation is for connector detection. The horizontal translation flexibility degree depends strongly on the processing capabilities of the hardware in order to meet the execution time criteria. For the prototype, the connector detection finally allowed a +10 degrees rotation of the ECU.

Distributing the processes on each of the six image processing devices from the system, an ECU can be analyzed in time so that the operator and the traceability application can execute their duties normally. Concluding after many tests, the IP-LC-FD system is executing a complete procedure under 6.5 s, and the minimum execution time was 3 s.

The distribution of tasks over four, respectively, six processing equipment was mandatory. The perspective in the images is always present for the used optical devices. The perspective could be eliminated using telecentric lenses, but the cost would have been highly affected. Additionally, the size of the analyzed ECU compared to the dimension of the pins would have been implied by probably more telecentric lenses, or even a moving board.

Another aspect that is worth mentioning is related to neural networks. Some of the implemented algorithms implied a high degree of neural network usage. None of these algorithms was able to meet the execution time criteria, due to the hardware and implicitly processing limitations. It is interesting to mention that the mentioned algorithms were providing the worst results even for the detection itself.

To avoid configuring the test parameters, a variant based on machine learning can be considered. This would require more complicated computing equipment and would increase costs, and changing the tested products is not done very often, which does not justify making such an investment.

An important issue that has to be discussed is related to lighting. For the experimental model, the diffuse lighting was enough for good results. In the case of the prototype, an important issue occurred: the equipment was placed in a large, artificially illuminated factory, and manipulated by operators. In this context, the diffuse lighting of the IP-LC-FD together with proper parameterization of the system, assured the correct detection, but some tests on a particular set of boards implied the use of additional side lighting, because consistent shadowing was detected.

For the experimental model, the detection rate was 95%, as required by the company.

The key performance indicators obtained for the prototype after testing and validation were highly consistent. All the modules and procedures were tested, obtaining more than a 98% success rate. The solution proved to be robust, the result of the capability test (repeatability) being 100% success.

## Figures and Tables

**Table 1:** Other results of the testing procedure.

Test Procedure	Success Rate
Testing the informational flow at the algorithm level (“fairness” concept). (a) All module/function activation signals producing the expected outcome (e.g., image capturing on the master/slaves generates the image file, the EdgeDetection function always determines the corresponding execution, the ImageDifference executes always the correct code, etc.); (b) correct transition between the states, no unknown state; (c) the application is providing outputs and allows the transition to the new cycle both in normal and debug functioning regimes.	100%
Verifying the communications and the threads. (a) The communication between the master and the slaves; (b) The communication with the traceability application; (c) Correct functioning of the local threads.	100%
Testing the local reporting module.	100%
Testing the ability to learn new types of boards and the management of saved ECUs.	100%
Verifying the data aggregation and integration from all processing equipment, and the concluding manner.	100%
Testing the referencing procedure (references addition, adjustments, etc.)	100%
Testing the correct connector detection and fault detection for small and large pins, clips, cracks.	98%
Testing the missing pins detection.	98%
Testing the extra pins detection.	98%
Capability Pass repeatability test: A good ECU is tested successfully 50 times in the industrial environment and 50 times the system provides the same result, Passed.	100%
